# Can brief telephone interventions reduce caregiver burden and depression in caregivers of people with cognitive impairment? - long-term results of the German day-care study (RCT)

**DOI:** 10.1186/s12877-019-1207-y

**Published:** 2019-07-25

**Authors:** Carolin Donath, Katharina Luttenberger, Elmar Graessel, Jennifer Scheel, Anna Pendergrass, Elisa-Marie Behrndt

**Affiliations:** Center for Health Services Research in Medicine, Department of Psychiatry and Psychotherapy, University Clinic Erlangen, Friedrich-Alexander-University Erlangen-Nürnberg (FAU), Schwabachanlage 6, 91054 Erlangen, Germany

**Keywords:** Day-care, Dementia, MCI, Informal caregivers, Telephone intervention

## Abstract

**Background:**

Day-care and telephone counseling have been discussed as effective support measures for caregivers of people with cognitive impairment.

**Methods:**

In a two-arm cluster-randomized trial involving multicomponent therapy for cognitively impaired persons in day-care centers and telephone counseling for their caregivers versus treatment as usual (TAU), we investigated long-term effects on caregivers’ burden and depressiveness. Person-caregiver dyads involving home-dwelling persons with MCI, mild dementia, or moderate dementia were eligible. Day-care centers were randomized into an intervention group (IG) or a control group (CG). Outcome assessors were blinded. Out of 359 caregivers who had completed a 6-month intervention phase (n_IG_ = 205, n_CG_ = 154), a total of 304 of them were available at the 12-month follow-up (n_IG_ = 173, n_CG_ = 131). Instruments for assessing were the Burden Scale for Family Caregivers – short version (BSFC-s) (caregiver burden) and the Well-Being Index Score (WHO-5) (depressiveness). Mixed ANOVAs were used for the main analyses; descriptive statistics and subgroup analyses were additionally performed; secondary analyses involved multiple linear regressions for the main outcomes that were significant in the unadjusted main analysis.

**Results:**

At follow-up, crude mean differences showed a nonsignificant advantage for the IG in caregiver burden [IG: −.20 (SD = 5.39) vs. CG: .76 (SD = 5.49), *p* = .126, d = .177] and depressiveness (reverse scored) [IG: −.05 (SD = 5.17) vs. CG: −.98 (SD = 5.65), *p* = .136, d = .173]. For caregiver burden, a mixed ANOVA resulted in significant main effects of group (F (1, 302) = 4.40; *p* = .037) and time (F (1.88, 568.96) = 3.56; *p* = .032) but not a significant interaction. The largest effects were found for the “mild dementia” subgroup (d = .443 for caregiver burden and d = .520 for depressiveness).

**Discussion:**

Positive long-term effects of a combined intervention involving telephone counseling for caregivers and multicomponent activation for patients were observed especially for mild dementia. However, the treatment effects washed out after the intervention ended.

**Trial registration:**

ISRCTN16412551 (date: 30 July 2014, retrospectively).

## Background

An estimated 35.6 million people worldwide were affected by dementia in 2009. This number is anticipated to increase to 66 million by 2030 and 115 million by 2050 [1]. Demographic change along with increases in life expectancy have led to the challenge that cognitive impairment and society’s handling of it are becoming a substantial issue for health care systems but also for the affected people and their caregivers [1–4].

There are multiple effective offers of support for people with cognitive impairment and their caregivers [5–8]. One of them is day-care, where “the patient is cared for with other dementia patients for up to eight hours a day by professionals [ …]. In some instances, non-pharmacological therapies to promote cognitive and everyday practical skills are offered” [9], and it has been recognized as an effective caregiver relief service [10]. Furthermore, attending day-care also seems to be associated with a higher quality of life for the persons with cognitive impairment (PCIs) [11].

Besides day-care as a care and relief service offered as part of routine health care, telephone counseling for caregivers of people with dementia has also been identified as an effective support measure especially with respect to depressiveness [12]. A recent systematic review concluded that counseling interventions for caregivers of people with dementia comprising various elements of psychoeducation, peer support, and skills training had the highest potential to improve caregiver outcomes [13].

In the German day-care study (DeTaMAKS), a multicomponent intervention for PCIs involving a two-arm randomized controlled trial (RCT) was implemented in 34 German day-care centers [14, 15]. In addition, an outreach telephone counseling intervention for their caregivers was carried out, and both were analyzed for their efficacy. The DeTaMAKS short-term effects were published [15, 16]. However, it is not yet clear whether the German day-care study interventions have long-term effects, and if so, for which aspects. A study from the Netherlands also investigated the effects of community-based day-care in combination with caregiver support; however, this study concentrated solely on outcomes related to the person with dementia [17].

The first aim of this study was to analyze whether the interventions aimed at the caregivers in the German Day-Care study (brief telephone counseling) that have been shown to be effective for a subsample in the short-term [18] could improve caregiver burden and/or depressiveness 1 year after the study began and 6 months after the end of the controlled study period.

### Hypothesis 1

Caregivers in the intervention group will show more favorable values in caregiver burden and depressiveness than caregivers in the control group 12 months after the study began.

A further aim was to analyze whether subgroup-specific intervention effects according to the severity of cognitive impairment would be evident in the long-term.

### Hypothesis 2

Depending on the cognitive status of the care recipient, the effects will differ. The most favorable effects will hold for the group of caregivers caring for people with mild dementia.

## Methods

### Study design

The DeTaMAKS study (dementia – day-care – MAKS therapy; ISRCTN16412551), which began in October 2014 and ended in March 2017, was designed as a cluster-randomized, controlled, two-arm prospective longitudinal study. We used a waitlist control group design, which meant that during the 6-month intervention phase, caregivers in the day-care centers in the intervention group received a brief telephone intervention, whereas the person in their care received the non-pharmacological MAKS therapy at the day-care centers. The dyads comprised of the caregiver and the person with cognitive impairment (PCI) in the control group received no project-specific intervention during this period. After the 6-month intervention phase, the day-care center staff who worked with the control group also received training in MAKS therapy. The brief telephone intervention ended in the intervention groups after 6 months. No caregiver received telephone counseling initiated by the study team between 6 months follow-up and 12 months follow-up. There were also data collected further 12 months later, which are planned to be published by Pendergrass et al.

All study participants had the option to take part in any additional support services that were offered by the German Health Care System. The Ethics Committee of the Medical Faculty of Friedrich-Alexander-University Erlangen-Nuremberg examined and approved all procedures before the beginning of the study (Ref. 170_14 B). Thirty-four participating day-care centers were stratified by study region. By drawing lots, they were randomly assigned to the intervention or control group at baseline. All day-care center users were screened to ensure they met the inclusion criteria for the study. The main inclusion criterion for the PCIs was an existing cognitive impairment that met the threshold for Mild Cognitive Impairment (Mini-Mental State Examination (MMSE) > 23 and a Montreal Cognitive Assessment (MoCA) score ≤ 22) or mild to moderate dementia (MMSE between 10 and 23). PCIs who fulfilled all inclusion criteria were included in the study if their informal caregiver also agreed to participate in the project (please see the study protocol for all inclusion criteria and more details about the study design [14]). The caregiver had to provide home care without payment for the PCI but did not need to be a relative. All included caregivers and PCIs gave their written informed consent and were free to leave the study at any time. According to the Medical Research Council’s (MRC) Framework to design and evaluate complex interventions [19] this trial is in the Phase III (evaluation) since a fully defined intervention is evaluated using a protocol in a controlled study with appropriate statistical power. According to an update of the MRC’s framework “cluster-randomized trials are one solution to the problem of contamination of the control group” in evaluation complex interventions [20].

### Sample

**Fig. 1:**
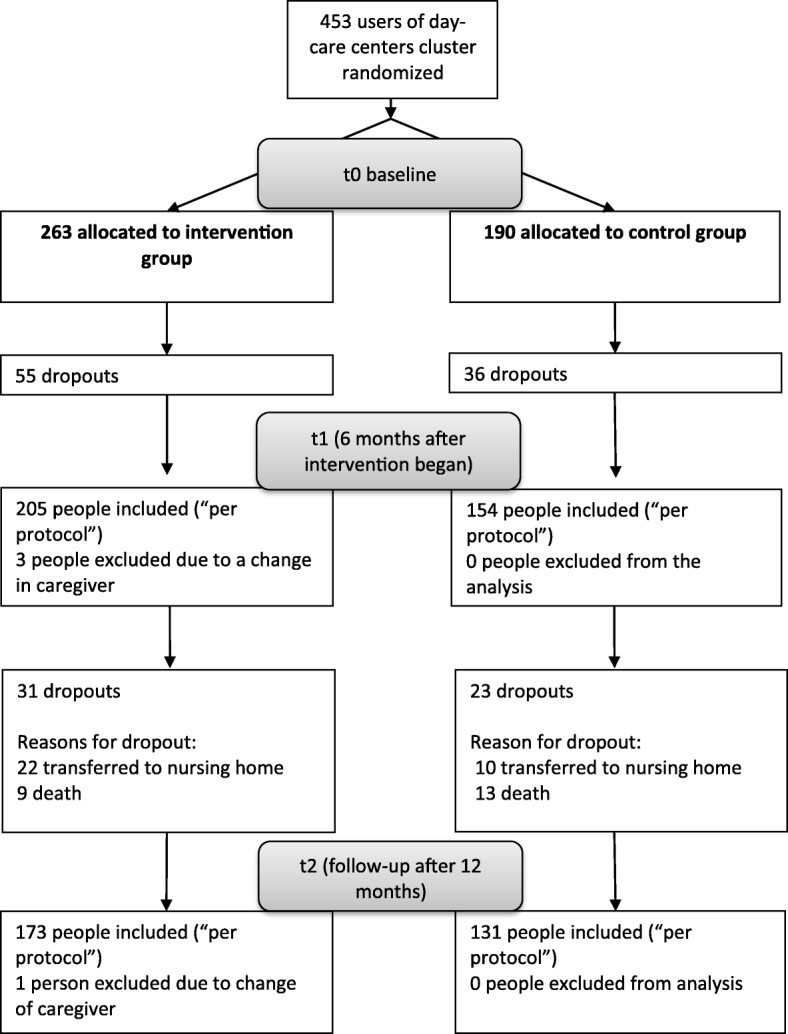
Flowchart depicting the composition of the samples

At the beginning of the study, 453 dyads consisting of a PCI and a caregiver were enrolled and allocated to the control or the intervention group depending on the cluster-randomization procedure. After 6 months (t1), a total of 359 (79.2%) participants (dyads) had completed the intervention period. The participants from 2 day-care centers had to be excluded during the intervention phase (first 6 months)—one because of insufficient administering of the intervention according to the manual, one because of a self-chosen termination of the collaboration agreement with the study center. For detailed reasons for the dropout and the distributions of the dropouts in the control and intervention groups between baseline and the 6-month follow-up, see Behrndt el al. [18]. Three hundred four dyads could be included in the analysis of the follow-up data after 12 months (t2). A total of 15.1% of participants had dropped out of the intervention group, and 14.9% had dropped out of the control group. Figure 1 presents the reasons for the dropout. The main reason was institutionalization (59.3%). One person had to be excluded from the analysis due to a change in caregiver.

The sample characteristics are depicted by group in Table 1. There was no statistically significant difference at the start of the study (t0) in any of the analyzed variables in the sample available at the 1-year follow-up (*N* = 304). The caregivers had a mean age of 59.5 years (SD = 11.6) ranging from 25 to 87. The persons with cognitive impairment had a mean age of 81.1 (SD = 7.5) ranging from 46 to 99, and 62.2% of them were women. Three-fourths (74.7%) of the caregivers were women, and about half of all caregivers were working (53.0%). The majority of them were the main caregiving person for the cognitively impaired (89.5%), and almost half of them lived together (46.4%). About two-thirds of the caregivers were children or in-laws (66.4%), and more than three-fourths were either married or living with a relationship partner (77.6%).

**Table 1:** Baseline characteristics of participants of the German Day-Care Study for the sample with 12-months follow-up data (*n* = 304)

Characteristics	Intervention group	Control group	Test for group differences
	(*n* = 173)	(*n* = 131)	(*p*)
*Caregiver (CG)*			
Age, *M* (*SD*)	60.0 (11.8)	59.0 (11.3)	.463^a^
Women, no. (%)	128 (74.0)	99 (75.6)	.753^b^
Educational attainment (yrs.), *M* (*SD*)^i^	10.8 (2.8)	10.8 (2.8)	.809^a^
Occupation: Employed, no. (%)	89 (51.4)	72 (55.0)	.543^b^
Marital status, no. (%)			.755 ^b^
Married/long-term relationship	133 (76.9)	103 (78.6)	
Widowed/Divorced	24 (13.9)	19 (14.5)	
Single	16 (9.2)	9 (6.9)	
Relationship to care recipient, no. (%)			.846 ^b^
Spouse	50 (28.9)	36 (27.5)	
Son/daughter (in-law)	113 (65.3)	89 (67.9)	
Others	10 (5.8)	6 (4.6)	
Caregiver burden (BSFC-s), *M* (*SD*)	11.7 (8.0)	13.0 (7.5)	.157^a^
Depressiveness (WHO-5), *M* (*SD*)	12.0 (6.0)	12.0 (5.6)	.799^a^
Benefits (BIZA-D), *M* (*SD*)	12.7 (4.9)	12.5 (5.5)	.747^a^
Health-related quality of life (EQ-5D-5 L), *M* (*SD*)	0.9 (0.2)	0.8 (0.2)	.140^a^
*Person with cognitive impairment (PCI)*			
Age, *M* (*SD*)	81.1 (7.4)	81.0 (7.6)	.843^a^
Women, no. (%)	107 (61.8)	82 (62.6)	.894^b^
Educational attainment (yrs.), *M* (*SD*)	9.8 (2.5)	9.6 (2.2)	.566^a^
Cognitive impairment (MMSE), *M* (*SD*)^ii^	19.7 (4.7)	19.7 (4.7)	.931^a^
Mild cognitive impairment	25.8 (1.5)	26.2 (1.5)	.215^a^
Mild dementia	20.5 (1.8)	20.5 81.7)	.986^a^
Moderate dementia	14.5 (2.4)	14.9 (2.0)	.349^a^
Activities of daily living (ETAM), *M* (*SD*)	18.0 (7.0)	17.8 (7.4)	.881^a^
Social behavior (NOSGER), *M (SD)*	15.5 (4.2)	15.5 (4.4)	.943^a^
Neuropsychiatric symptomatology (NPI), *M (SD)*	5.0 (2.7)	5.3 (2.7)	.272^a^
Care level, no. (%)^iii^			.408^b^
None	7 (4.0)	12 (9.2)	
0	16 (9.2)	13 (9.9)	
1	95 (54.9)	63 (48.1)	
2	53 (30.6)	42 (32.1)	
3	2 (1.2)	1 (0.8)	
Use of anti-dementive medication, no. (%)^iv^	120 (69.4)	98 (74.8)	.297^b^
*Care situation*			
Main caregiver, no. (%)	153 (88.4)	119 (90.8)	.499^b^
Sole informal caregiver, no. (%)	97 (56.1)	70 (53.4)	.648^b^
Living together, no. (%)	82 (47.4)	59 (45.0)	.683^b^
Duration of informal care (month), *M (SD)*	61.5 (50.1)	60.3 (55.1)	.846^a^
Frequency of day-care use per week, *M (SD)*^v^	1.9 (1.2)	1.9 (1.1)	.809^a^
Informal care time per day (h), *M (SD)*^vi^	3.0 (2.1)	3.1 (2.0)	.538^a^
No. of additional offers of formal care support, *M (SD)*^vii^	1.6 (1.3)	1.6 (1.3)	.761^a^
Use of other offers of caregiver counseling (%)^viii^	44 (25.4)	47 (35.9)	.058^b^

^i^ Minimum: 7 yrs. (no compulsory school-leaving certificate) – Maximum: 18 yrs. (university degree)

^ii^ Mild Cognitive Impairment: MMSE 30–24, Mild dementia: MMSE 23–18, Moderate dementia: MMSE 17–10

^iii^ Extent to which nursing care is needed according to the German Health and Care Insurance: none (no needs), 0 (low needs), 1 (moderate needs), 2 (high needs), and 3 (very high needs)

^iv^ Intake of memantine or acetylcholinesterase inhibitors

^v^ Average frequency per week in the first month

^vi^ Hours of average informal care per day adjusted for day-care attendance at baseline

^vii^ Sum index of (in addition to day-care) formal care and support offers used (care service, care group, meals on wheels, respite care, ambulatory care service, home help service)

^viii^ Displays the use of either one: caregiver skill training/counseling service for caregivers/support group for caregivers at the beginning of the study

^a^ t-Test/U-Test

^b^ Chi-square Test

### Intervention

Caregivers from the intervention group received a brief outreach telephone intervention during the 6 months after baseline. The contents of three telephone calls were based on a manual designed specifically for the study. We incorporated tested procedures from stress psychology into the manual to fit the caregivers’ situations [21]. Psychoeducative information regarding dementia and dealing with dementia-specific challenging behaviors were also implemented [22, 23]. Each call lasted up to 1 h. The counselors had received training especially for the intervention and had the main task of supporting the caregivers in developing strategies for self-management [24]. Other goals were helping to reduce the stress due to home care and to help the caregivers deal with challenging behaviors. By improving the caregivers’ skills, the aim of the intervention was to “empower” them. For more detailed information about the telephone-based caregiver intervention and a detailed description of the procedure, see the published study protocol [14] and the report of the immediate effects [18]. After 6 months, telephone support from the study team stopped. Caregivers in both the control and intervention groups were free to use offers of support such as caregiver counseling from the German Health Care System.

During the 6-month intervention period, PCIs in the intervention group also received the multicomponent, non-pharmacological MAKS Therapy [15, 25, 26], which was provided by the day-care centers. MAKS therapy consisted of motor stimulation, the encouragement of activities of daily living, and cognitive stimulation in a social setting. After the 6-month intervention phase, the day-care centers of the intervention and control groups were free to offer MAKS therapy but without project-specific support or reimbursement.

### Assessments

Via computer assisted telephone interviews (CATIs), all caregiver were assessed once at baseline, at the end of the intervention period (after 6 months), and again after six more months (12 months after baseline) regarding the caregiving situation (self-rating), the PCIs’ situation (observer-based rating), and the care situation.

#### Primary outcomes

##### The Burden Scale for Family Caregivers short (BSFC-s [27, 28])

The BSFC-s assesses informal caregivers’ subjective burden. Higher values indicate greater burden.

##### WHO-5 Well-Being Index (WHO-5 [29, 30])

This measures well-being in terms of level of depressiveness by evaluating a person’s mood during the last 14 days (lower values indicate greater depressiveness). The WHO-5 is used as a screening tool for unipolar depression [31]. Here, values are interpreted in the sense of depressiveness.

#### Potential predictors of primary outcomes

Socio-demographic variables including the age and sex of the caregivers were assessed. The study group was documented as well.

##### MAKS therapy available in day-care for the last 6 months (t1-t2)

One year after baseline, all day-care centers were asked if they had administered MAKS therapy in the last 6 months after the end of the intervention phase (yes/no). This was used as an indicator of whether the day-care centers had implemented therapy that went beyond treatment as usual (TAU) and was targeted at the PCI.

##### Frequency of day-care use (t0-t1)

In the 6-month intervention phase, the day-care centers were asked monthly about the PCIs’ average frequency of day-care use per week (months 1–6).

##### The Erlangen Test of Activities of Daily Living in Persons with Mild Dementia or Mild Cognitive Impairment (ETAM, t0 [32, 33])

The ETAM is an objective performance test designed to measure the PCIs’ abilities in activities of daily living. Scores range from 0 to 30, with higher values indicating better abilities to perform ADL. The assessment was conducted at the day-care centers by staff who were trained at the study headquarters.

##### Informal care time per day (h; t0)

The subscale of the Resource Utilization in Dementia (RUD [34]) questionnaire that includes estimations of time in terms of ADL and IADL was pooled into one question (In the last four weeks, how many hours per day did you or other persons (relatives, friends) spend on average actively helping the person in your care with these activities (e.g. going to the toilet, grooming, taking medication, housekeeping).

##### Major adverse event in the care of the PCI between 6 months follow-up and 12 months follow-up (t2)

Caregivers were asked if one of three types of severe adverse events (falls resulting in injury, other injuries requiring medical treatment, and other serious adverse events) had happened to the person in their care (yes/no).

##### Major event in the life situation of the caregiver between 6 months follow-up and 12 months follow-up (t2)

Caregivers were asked if they themselves had experienced major events in their lives during the last six months (“Have there been any major events in your life/care situation during the last six months?”) with the response options “yes” or “no” and were also asked to identify the event.

##### Nurses’ Observation Scale for Geriatric Patients (NOSGER; t0 [35])

The “social behavior” subscale of the NOSGER was used in the CATIs to determine the PCIs’ social behavior from the viewpoint of the caregiver. Higher values show greater impairment.

##### Neuropsychiatric Inventory Questionnaire (NPI-Q; t0 [36])

The NPI was administered to ask caregivers to evaluate the PCIs’ neuropsychiatric symptoms in the form of screening questions (yes/no) concerning twelve symptom domains.

The number of additional kinds of formal care support that were used (t0) and the use of other opportunities to obtain caregiver counseling (t0) were assessed with the adapted Resource Utilization in Dementia (RUD [34]) questionnaire and the Questionnaire for the use of medical and non-medical services in old age (“Fragebogen zur Inanspruchnahme medizinischer und nicht-medizinischer Versorgungsleistungen im Alter”, FIMA [37]), which evaluates the use of resources by both the PCIs and their informal caregivers.

### Statistical analysis

The analyses were computed with IBM SPSS Statistics 21. Descriptive statistics are provided as basic information. T-Tests for independent samples were used to test for significant differences, and Cohen’s d was calculated to obtain an effect size for group differences.

For the main analysis, in order to explore the effects of the RCT on the dependent variables, caregiver burden and depressiveness (Hypothesis 1), two mixed ANOVAs (mANOVA) with repeated measures were carried out. The within-subject variable was time; the between-subject variable was group (intervention versus waitlist control). To avoid over-interpretation of “unadjusted” effects in the main analysis, we determined a priori to carry out another analysis (secondary analysis) in which we controlled for other potential predictors of the dependent variable. This secondary analysis involved multiple linear regressions with either caregiver burden or depressiveness as the dependent variable and different caregiver-related constructs as well as health care system utilization variables as predictors. The secondary analysis was applied only to outcomes that were significant in the main analysis. Potential predictors were checked for multicollinearity before being included in the multiple regressions.

We computed difference scores (t2-t0) for the two main outcomes caregiver burden and depressiveness and compared them across the subgroups that were defined by the different levels of severity of the PCIs’ cognitive impairment (Hypothesis 2): Mild Cognitive Impairment (MCI), mild dementia, and moderate dementia. T-Tests and Cohen’s d were again used to provide interpretations of differences between the subgroups.

Since the matter concerns a cluster-randomized trial we report according to the CONSORT-suggestions the Intraclass correlation coefficient (ICC), which is .046 in this study. There were 34 daycare centers randomized, 17 to each study arm. The mean number of available places in the participating day-care centers was 26.8 for the control and 24.2 for the intervention group (t = .279; *p* = .782). The mean number of occupied places in the day-care centers was 15.6 for the control and 16.8 for the intervention group (t = −.561; *p* = .579). We compared the frequency distributions of recruited dyads between control and intervention group, there was also no significant difference (Chi^2^ = 15.333; *p* = .500).

## Results

### Caregiver burden – hypothesis 1

#### Descriptives

Table 2 presents the means and standard deviations for caregiver burden in both groups at each time point. While descriptive statistics depicted a worsening of caregiver burden from t0 to t2 in the control group (Mean Delta = +.76 (SD = 5.49)) versus a small improvement in the intervention group (Mean Delta = −.20 (SD = 5.39)), the difference was not significant (*p* = .126). The effect size of the crude difference values (d = 0.18) was somewhat below a small effect according to Cohen (Cohen, 1988).

**Table 2:** Descriptive statistics for the dependent variables caregiver burden/depressiveness by group for the three measurement points (N = 304)

Variable		Descriptives			t-Test for independent samples		Effect size for mean difference
		Mean	Standard Deviation	ΔM (SD) (T2-T0)	t (df)	*p*-value	Cohen’s d [95%-CI]
Caregiver Burden							
Intervention group (*N* = 173)	T0	11.71	8.00	−.20 (5.39)^a^	t (302) = 1.53	.126	0.177 [−0.051; 0.406]
	T1	10.93	7.77				
	T2	11.50	8.26				
Control group (*N* = 131)	T0	12.98	7.48	.76 (5.49)^a^			
	T1	12.83	7.90				
	T2	13.75	8.24				
Depressiveness (reverse coded)							
Intervention group (*N* = 173)	T0	11.90	6.03	−.05 (5.17)^b^	t (302) = 1.49	.136	0.173 [−0.056; 0.401]
	T1	12.48	5.92				
	T2	11.85	5.96				
Control group (*N* = 131)	T0	12.07	5.61	−.98 (5.65)^b^			
	T1	11.69	5.66				
	T2	11.09	6.04				

^a^Positive values depict a worsening in the development of the outcome

^b^Negative values depict a worsening in the development of the outcome

#### Main analysis

**Fig. 2:**
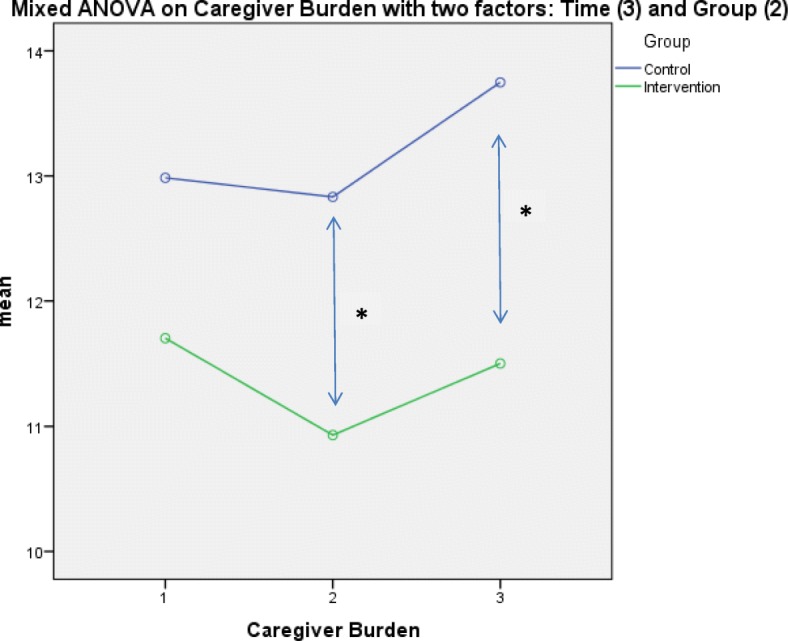
Depiction of the main effects of caregiver burden with within- and between-subject factors. Legend: * significant difference, *p* < .05

A mixed ANOVA with the dependent variable caregiver burden, the within-subject variable “time” (3-fold: therapy start, therapy end, 12-month follow-up) and the between-subject factor “group” (2-fold: intervention group, control group) yielded a significant main effect of time (Greenhouse-Geisser-corrected): F (1.88, 568.96) = 3.56; *p* = .032; partial η^2^ = .01, indicating that caregiver burden changed in the total sample over time. There was also a significant main effect of group: F (1, 302) = 4.40; *p* = .037; (no partial η^2^ because there were fewer than 3 groups) illustrating a significant difference in caregiver burden (which did not exist at the beginning of the study – see Table 1) between the intervention and control group. It was investigated at which time-point the group differences were responsible for the significant group effect in the mANOVA. For both time points, right after the intervention ended (t1) as well as 6 months after the intervention end (t2) were the caregiver burden values significant lower in the intervention group than in the control group: t (302) = 2.10; p = .037 for t1 and t (302) = 2.35; *p* = .019 for t2. Figure 2 shows descriptively that caregiver burden fell somewhat more steeply during the intervention period in the intervention group compared with the control group. During the follow-up period, caregiver burden increased in both groups, but the increase was steeper in the control group. Statistically, there was no significant interaction effect for caregiver burden between the two factors group and time observed: F (1.88, 568.96) = 1.51; *p* = .223; partial η^2^ = .005.

#### Secondary analysis

After the multicollinearity analysis of the potential predictors, the variable *occupation of the caregiver* was omitted from further analysis because of an association with *caregiver’s age* (r = −.647). Furthermore, the *cognitive impairment* (MMSE) and *Activities of daily living* (ETAM) measures were substantially correlated (r = .624). Because the association with the dependent variable was higher in the ETAM than in the MMSE, the ETAM was retained for the regression analysis. A significant model for the multiple linear regression analysis with caregiver burden at the 12-month follow-up as the dependent variable was the result: F (13) = 8.48; *p* < .001. The proportion of variance explained by the model was 27.5% (R^2^). Significant predictors of caregiver burden 1 year after the start of the study were the age of the caregiver (*p* = .034), sex of the caregiver (*p* = .022), hours of average informal care per day without day-care time (*p* < .001), neuropsychiatric symptomatology (*p* < .001), and use of other offers for caregiver counseling at the beginning of the study (*p* < .001). Higher age and a female caregiver were associated with a higher burden. A greater need and use of informal care measured in hours was associated with a higher burden as well as a higher prevalence of neuropsychiatric symptoms in the person with cognitive impairment such as agitation, sleep disturbances, or anxiety plus the need for and use of other counseling services for caregivers already at the beginning of the study. The group variable (intervention vs. control) was not statistically significant in the secondary “adjusted” analysis. The results of the regression analysis are presented in Table 3.

**Table 3:** Secondary Analysis: Multiple linear regression analysis with caregiver burden after 12 months (T2) as the dependent variable (*n* = 304)

Caregiver Burden (12-month follow up = T2)						
					*95% CI*	
*Predictors*	*Unstand. ß*	*Standardized ß*	*T*	*p*	*Lower limit*	*Upper limit*
Group (CG/IG)	−1.462	−.087	−1.708	.089	−3.147	.223
Age caregiver	.081	.113	2.132	**.034**	.006	.156
Sex caregiver (0 = female, 1 = male)	−2.249	−.118	−2.301	**.022**	−4.172	−.326
MAKS therapy available in day-care for the last 6 months (t1-t2)	.093	.005	.098	.922	−1.775	1.961
Frequency of day-care use	−.194	−.024	−.476	.634	−.995	.607
Activities of daily living (ETAM)	−.025	−.022	−.407	.684	−.148	.097
Informal care time per day (h)	.749	.219	4.215	**<.001**	.399	1.099
Major adverse event in the care of the PCI in the last 6 months (0 = no, 1 = yes)	.957	.053	1.042	.298	−.851	2.765
Major event in the life situation of the caregiver in the last 6 months (0 = no, 1 = yes)	.886	.045	.865	.388	−1.130	2.902
Social behavior (NOSGER)	.117	.060	1.101	.272	−.092	.325
Neuropsychiatric symptomatology (NPI)	.671	.217	3.953	**<.001**	.337	1.004
No. of additional offers of formal care support	.436	.056	1.098	.273	−.345	1.218
Use of other offers of caregiver counseling (0 = no, 1 = yes)	3.591	.198	3.698	**<.001**	1.680	5.502

Significant *p*-values (<.05) are in bold.

*Abbreviations*: *BSFC-s* Burden Scale for Family Caregivers short (score), *CG* Control Group, *IG* Intervention Group, *MAKS-therapy* Multicomponent Therapy for cognitively impaired (motoric -, activities of daily living-, cognitive stimulation), *ETAM* Erlangen Test of Activities of Daily Living, *PCI* Person with cognitive impairment, *NOSGER* Nurses Observation Scale for Geriatric Patients, Subscale Social Behavior*, NPI* Neuropsychiatric Inventory

### Depressiveness– hypothesis 1

#### Descriptives

The means and standard deviations for depressiveness in both groups at each time point are reported in Table 2. Again, the descriptive statistics show a more intense worsening of the outcome from t0 to t2 – here depressiveness – in the control group (Mean Delta = −.98 (SD = 5.65)) versus stabilization in the intervention group (Mean Delta = −.05 (SD = 5.17)) (because it was operationalized as “well-being,” positive values here indicate improvement). The difference in the difference values between the intervention and the control group was not statistically significant (*p* = .136) with an effect size of d = 0.17, which can be interpreted as below the threshold of a small effect according to Cohen (Cohen, 1988).

#### Main analysis

**Fig. 3:**
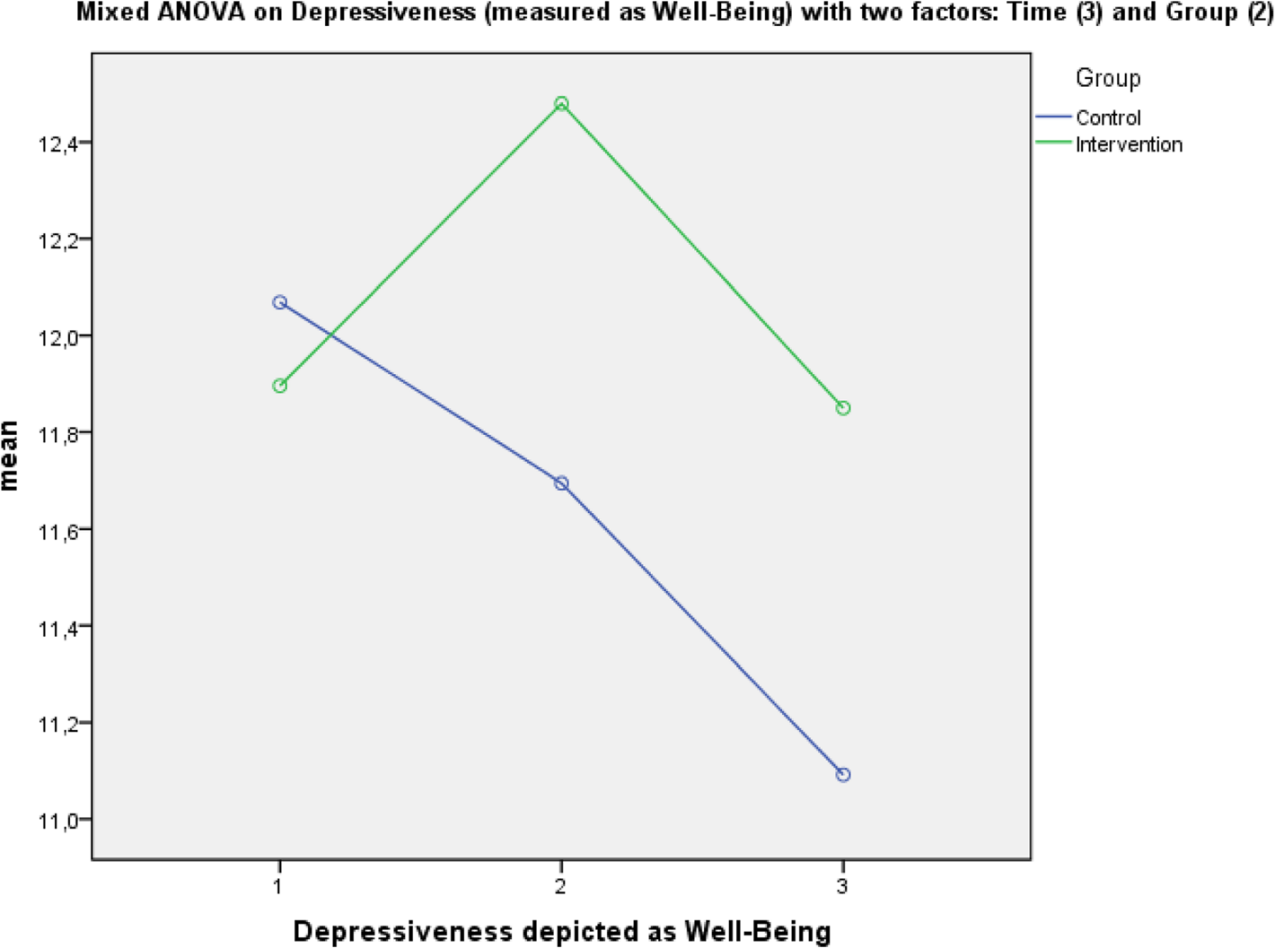
Main effects of depressiveness (reverse coded) with within- and between-subject factors

A mixed ANOVA with the dependent variable depressiveness, the within-subject variable “time” (3-fold: therapy start, therapy end, 12-month follow-up) and the between-subject factor “group” (2-fold: intervention group, control group) did not show a significant main effect of time (Greenhouse-Geisser-corrected): F (1.94, 587.18) = 2.45; *p* = .089; partial η^2^ = .01, which indicated that depressiveness did not show substantial change over time in the total sample. Also, no significant main effect of group was found: F (1, 302) = 0.60; *p* = .438. However, Fig. 3 shows graphically that depressiveness (measured as “well-being”) developed in the clinically desired direction in the intervention group during the intervention period (T0-T1), whereas in the control group, depressiveness rose (well-being sunk) across the entire observation period. The graphical result suggests that in the intervention group, depressiveness rose again (and well-being decreased) after the intervention period ended until the 12-month follow-up. No statistically significant interaction between the two factors group and time could be observed for depressiveness: F (1.94, 587.18) = 1.67; *p* = .189; partial η^2^ = .01.

#### Secondary analysis

A secondary analysis was not carried out for depressiveness because the main analysis did not yield significant effects.

### Subgroup analysis– hypothesis 2

An analysis of differences between subgroups defined by the severity of cognitive impairment was carried out by analyzing difference scores between the 1-year follow-up and the start of the study in caregiver burden and depressiveness.

**Fig. 4:**
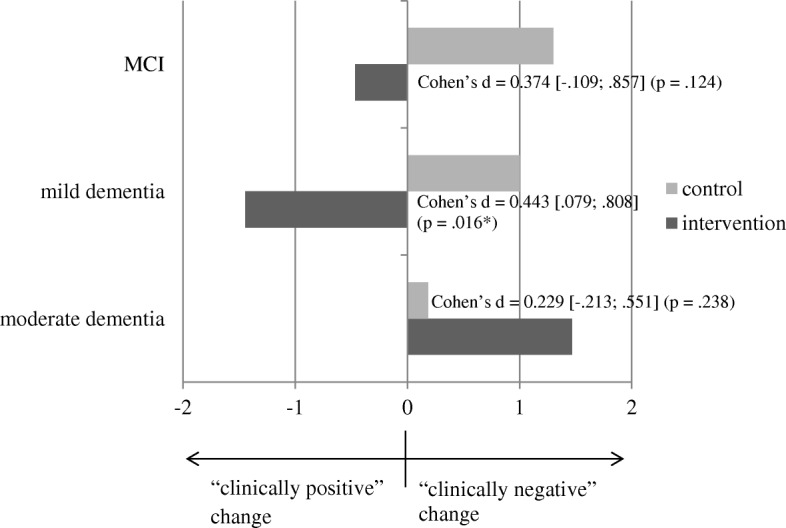
Illustration of effects based on difference scores (t2-t0) for subjective caregiver burden – by subgroups characterized by different levels of severity of cognitive impairment

For caregiver burden, a significant small-sized effect (d = .443; *p* = .016) was observed in the group of caregivers who cared for persons with mild dementia. In the group of caregivers with a relatively mildly cognitively impaired person, the effect was descriptively favorable for the intervention group (d = .374) but was not significant (*p* = .124). However, caregivers who cared for a person with moderate dementia experienced – descriptively – a more intense worsening of burden when in the intervention group 1 year after the beginning of the study (and 6 months after the intervention had ended) compared with the control group, but again, this effect was not statistically significant (*p* = .238). See Fig. 4 for a graphical representation. The mean difference scores and corresponding t-statistics are included in Table 4 for each group.

**Table 4:** Subgroup-specific descriptive statistics for change in the dependent variables caregiver burden/depressiveness by the severity of cognitive impairment (*n* = 304)

Variable		Descriptives		t-test for independent samples		Effect size for mean difference
		Mean Diff. (T2-T0)	Standard Deviation	t (df)	*p*-value	Cohen’s d [95% CI]
*Caregiver Burden*	*Severity*					
IG (*N* = 41)	MCI	−.46^a^	4.67	t (69) = 1.56	.124	0.374 [−0.109; 0.857]
CG (*N* = 30)	MCI	1.30^a^	4.77			
IG (*N* = 72)	mild	−1.44^a^	5.07	t (122) = 2.44	**.016**	0.443 [0.079; 0.808]
CG (*N* = 52)	mild	1.00^a^	6.09			
IG (*N* = 60)	moderate	1.47^a^	5.87	t (107) =−1.19	.238	0.229 [−0.611; 0.154]
CG (*N* = 49)	moderate	.18^a^	5.28			
*Depressiveness (inversely coded)*	*Severity*					
IG (*N* = 41)	MCI	.46^b^	6.08	t (69) =−.61	.542	0.147 [−0.620; 0.329]
CG (*N* = 30)	MCI	−.30^b^	3.63			
IG (*N* = 72)	mild	.76^b^	4.69	t (122) =−2.86	**.005**	0.520 [−0.886; −0.154]
CG (*N* = 52)	mild	−1.92^b^	5.77			
IG (*N* = 60)	moderate	−1.37^b^	4.88	t (87.88) = .88	.382	0.169 [−0.213; 0.551]
CG (*N* = 49)	moderate	−.39^b^	6.43			

Significant *p*-values (<.05) are in bold

^b^Negative values depict a worsening in the outcome

Positive values depict a worsening in the outcome

**Fig. 5:**
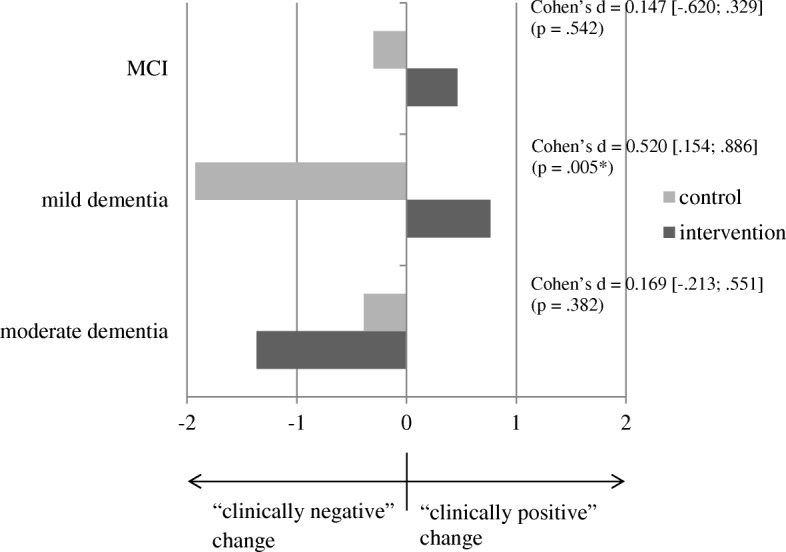
Illustration of effects based on difference scores (t2-t0) for depressiveness (operationalized as “well-being”) – by subgroups characterized by different levels of severity of cognitive impairment

For depressiveness (see Fig. 5), the pattern was basically the same: a significant moderate-sized effect (d = .520; *p* = .005) was observed in the group of caregivers who cared for persons with mild dementia. A descriptively favorable but nonsignificant (*p* = .542) effect for the intervention group (d = .147) was seen in caregivers of persons with MCI. Again, an statistical non-significant reversal of the effect was seen in the group of caregivers of people with moderate dementia: There was a seemingly (d = 0.169) more intense worsening of depressiveness in the intervention group 6 months after the end of the intervention compared with the control group, but this change was not statistically significant (*p* = .382). See Table 4 for mean difference scores and the corresponding t-statistics.

## Discussion

The aim was to analyze whether a brief telephone intervention aimed at the caregivers of cognitively impaired persons would display long-term effects on caregivers’ burden or depressiveness 1 year after the intervention had begun (and 6 months after it had ended). Purely descriptively, a favorable but nonsignificant development in the outcomes was observed: Whereas caregiver burden and depressiveness had risen in the one-year period in the control group, both remained essentially stable in the intervention group.

The main analysis (mANOVA) showed that caregiver burden changed significantly over time and that the groups differed significantly over the time period of the study (after equivalency had been established before the intervention). The progress of the outcome showed differences but also similarities: While in the first 6 months (the intervention period), caregiver burden decreased in the intervention group and remained stable in the control group, during the second 6 months (TAU for both groups), caregiver burden increased in both groups, but the increase was steeper in the CG. For various predictors, in the adjusted analysis, “group” was not a predictor of caregiver burden after 1 year, but the demographics of the caregiver, a larger amount of informal caregiving time because of higher needs, an evident neuropsychiatric symptomatology (i.e. non-cognitive symptoms) in the care recipient, and the need for and use of additional opportunities to obtain caregiver counseling. Thus, in other words, caregiver burden after 1 year was associated with high (time-related) demands of care, possibly caused by non-cognitive neuropsychiatric symptoms, such as wandering or sleep disturbances, at the beginning of the study, and already being in need of support from counseling and using such offers at the beginning of the study. The status of how burdened the caregivers were at the beginning of the study had strong predictive value for later levels of caregiver burden and was superimposed on possible positive influences of an additional brief caregiver counseling intervention.

For depressiveness, no significant long-term effects could be demonstrated when we looked at the whole sample. Visually, well-being seemed to increase slightly (depressiveness decreased) in the intervention group during the intervention period (the first 6 months) before it reverted back to the baseline level after the intervention had ended (second 6 months). A different trajectory was found in the control group where a steady decrease in well-being (and thus an increase in depressiveness) took place during the entire 12-month period. However, there was no significant interaction effect, but rather only a descriptive effect of time that showed that depressiveness changed in both groups over time.

The second aim was to determine whether subgroup-specific effects of the intervention according to the level of cognitive impairment would be found in the long-term data. 1) In the subgroup of caregivers of people with mild dementia, there was a small effect for caregiver burden and a medium-sized effect for depressiveness that were detected after 12 months, favoring the intervention group in this subgroup. 2) In the subgroup of caregivers who cared for recipients with moderate dementia the 12-month measures of caregiver burden and depressiveness showed more intense declines in the former intervention group than in the control group. However, this finding was far from statistically significant. Nevertheless, one hypotheses might be that caregivers in the intervention group were especially disappointed that the intervention stopped at the moment they needed it most – when the cognitive deterioration of their relative proceeded being in an already higher stage of impairment at study start. Perhaps they had become accustomed to receiving counseling support, and then it stopped even though they were still in need. This may mean – as a practical implication – that it is urgent and important to have a (time-period-wise) longer telephone counseling phase. The caregivers in the control group might have organized themselves or used some kind of counseling support from the German Health System and did not have the experience of losing support that had previously been offered. We have also checked if there were specific selection effects that could be an explanation, this was not the case.

Before comparing the results of this study with the results of other studies involving telephone interventions for caregivers of people with dementia – one methodological difference has to be mentioned: Many studies have used a higher dose of the intervention than the study reported here in which 3 phone calls in 6 months were applied [38–41]: The intervention periods in other studies usually lasted 3 to 12 months, ranging from (in the majority: biweekly) a minimum of 6 in 3 months [42] to a maximum of 23 calls in 12 months [38]. There were two pilot studies with weekly calls (12 in 3 months: [43]); (8 in 2 months: [44]) with a weekly opportunity to participate [45]. Outcomes were mostly – as they were here – subjective burden and/or depressiveness. Most reports referred to post-treatment data, whereas only one explicitly referred to long-term effects [46] as we did. A recent systematic review [47] hinted that the dose and regularity of calls are crucial for effectiveness, that is, studies with more calls over a longer period of time had more favorable outcomes than studies with fewer but longer telephone calls.

There were also hints that especially studies with several elements in their telephone counseling intervention (e.g. psychoeducation, skill training) could lead to improvements [13]; the only study in the systematic review that was judged as having a low methodological risk and involved a telephone intervention had such a multicomponent concept and found improvement in depression as the caregiver outcome [41]. The brief telephone counseling intervention analyzed here was also a multicomponent intervention and included situational training, psychoeducation, assessment of the patient’s own health status, goal and problem analysis, and specific goal setting.

A Cochrane Review on the efficacy of telephone counseling for informal caregivers of people with dementia [12] concluded that the results were most trustworthy for the outcome of depression and that telephone counseling could reduce caregivers’ depressive symptoms. The evidence for caregiver burden was less certain. A meta-analysis of four studies showed a nonsignificant pooled difference in caregiver burden between the telephone counseling group and the control group. However, statistical significance in favor of burden was not quite achieved (*p* = .05), and the heterogeneity measure was relatively high (I^2^ = 45%). A further recent systematic review stated that in half of the analyzed studies (6 of 12), favorable effects on the outcome of depression were visible and concluded that the findings showed “mixed effects” [13]. Again, for the outcome of caregiver burden, just as Lins et al. [12] had found, Waller et al. [13] identified an even less clear possible beneficial effect (reduction of burden in 4 out of 11 studies).

In our study, positive intervention effects were found for caregiver burden (benefits for the intervention group demonstrated in the mANOVA and in inferential statistics on difference scores for the subgroup with “mild dementia”) but in general not for depression (benefits seen only in inferential statistics on difference scores in the subgroup with “mild dementia”). As in other studies [43, 45], we were able to report a descriptive reduction in burden for crude difference scores, but the reduction was not statistically significant.

In comparison with the one study reporting long-term effects [46], similarly, our study did not support effects of depressiveness in the long-term (measured with validated scales) for the whole sample. However, Wilz et al. [46] found an effect for emotional well-being assessed with a one-item self-rating. Also, just as Wilz et al. [46] had found, we found effects for subgroups in the long-term evaluation of caregiver outcomes. However, burden was not assessed in the previous study.

### Strengths and limitations of the study

This study has several strengths: First, we applied an RCT with as much blinding as possible when the psychosocial interventions were applied (assessments via computer-assisted telephone interviews (CATIs) with blinded assessors). At the same time, the results demonstrated high ecological validity because the interventions were applied in existing health care structures, that is, day-care centers. Second, the interventions were standardized, manualized, and published so that they could be assessed by other experts and avoid the “black-box phenomenon.” Third, the outcome measurement included well-researched outcomes that were operationalized with established and well-validated instruments. Fourth, the quality of the data was high because there were only a very small number of single missing values that needed to be imputed. Fifth, there was high transparency in the conducting and reporting of the study, that is, the study protocol was published [14], the study was registered, and a second research institution checked the data for plausibility.

However, there were also limitations that should be taken into account when interpreting the results. First, complete blinding was not possible – both the counselors and the participants were aware of who received the intervention – because the intervention concerned phone calls.

Second, outcome data were assessed via self-report. However, possible biases in self-reports (e.g. social desirability or regression toward the mean) would be likely to impact both the control and intervention groups.

Third, there is a noticeable dropout rate due to death or institutionalization, and this might be associated with a certain selection process (the most healthy and less affected care recipients and their caregivers remained in the sample). However, this also affected both the intervention and control groups. The baseline characteristics of the sample analyzed here were compared with the baseline characteristics (t0) of the sample analyzed by Behrndt et al. [18] to evaluate the short-term (6-month = t1) effects. If there was a substantial selection effect, the sample analyzed here (t0-t2) should have more favorable values in important constructs such as burden, cognitive status, ADL, and hours of care than the previously analyzed sample (t0-t1). However, the differences in the sample presented here were rather small to marginal. Subjective burden was 0.2 points lower in both the intervention group and the control group than in the t0-t1 sample. Cognitive status was the same in the intervention groups of the two samples and 0.4 points lower in the control group of the t0-t1 sample. For ADLs, the sample analyzed here was .2 (IG) or .7 (CG) points better than the t0-t1 sample. Finally, there was no difference in hours of care between the two samples in the intervention group, and in the control group, the t0-t2 sample had .2 fewer hours.

Fourth, the dropout rate led to a smaller N, which reduced the power of the statistical tests and may have contributed to the nonsignificant difference between the groups in the mean difference scores for the outcome, which displayed the expected direction of outcome development but failed to achieve statistical significance.

Fifth, the matter concerned a “combined intervention”: in the same period in which the telephone counselling was carried out, the individuals with cognitive impairment received a multi-component, non-drug therapy at the day-care centers. The patient-oriented intervention influenced cognition, activities of daily living and neuropsychiatric symptoms [15]. However, Behrndt et al. [18] in consequence checked whether a change in neuropsychiatric symptoms in the PCI during the therapeutic phase was a predictor for caregiver burden or caregiver depressiveness in the short-term results (right after the intervention stopped: t1). This was not the case. Behrndt et al. conclude: “on the whole, the effect of the caregiver intervention cannot be attributed to the change in PCIs’ neuropsychiatric symptoms” [18]. Thus it is implausible that this construct has significant predictive power for caregiver burden/depressiveness 6 months after the end of both intervention elements. Behrndt et al. suggest though, that “for a final clarification of a potential confounding effect of the MAKS therapy on the caregiver telephone intervention, a new study with separately administered single interventions is necessary” [18].

Sixth, since the intervention effect seemed to wash out as soon as the intervention stopped one needed for a more precise evaluation of the telephone counselling intervention in a future trial an active control group which fulfilled the condition of receiving attention. Only then it would be possible to differentiate whether the contents of the counselling or simply giving someone “support” was responsible for reduction of burden.

### Clinical implications

Brief telephone counseling for caregivers of PCIs who are attending day-care centers has the advantages of offering an inexpensive, low threshold, time-saving way to offer support for caregivers. The use of trained external counselors secured a quality standard and also time flexibility. However, the data suggest that the effects of this support fade fast as soon as the counseling stops. Caregiver burden and depressiveness increase when the caregiver is caring for a chronically progressive care recipient with cognitive impairment when the caregiver does not receive sufficient support. Thus, to maintain the positive effects of the telephone intervention, it seems urgently important to continue counseling.

## Conclusion

There are hints toward positive effects of brief telephone counseling for caregivers who are caring for people with dementia, especially concerning subjective caregiver burden. However, such effects are especially evident for caregivers of people with mild dementia and washed out after the intervention ended.

## Data Availability

Outputs of the data analysis are available from the authors upon reasonable request.

## Abbreviations

BSFC-s: Burden Scale for Family Caregivers – short version
CATIs: Computer assisted telephone interviews
CG: Control Group
CONSORT: Consolidated Standards of Reporting Trials
DeTaMAKS: German day-care study
ETAM: Erlangen Test of Activities of Daily Living in Persons with Mild Dementia or Mild Cognitive Impairment
FIMA: Questionnaire for Health-Related Resource Use in an Elderly Population
ICC: Intraclass correlation coefficient
IG: Intervention Group
mANOVA: mixed Analysis of Variance
MCI: Mild Cognitive Impairment
MMSE: Mini Mental Status Examination
MoCA: Montreal Cognitive Assessment
MRC: Medical Research Council
NOSGER: Nurses’ Observation Scale for Geriatric Patients
NPI-Q: Neuropsychiatric Inventory-Questionnaire
PCIs: Persons with cognitive impairment
RCT: Randomized controlled trial
RUD: Resource Utilization in Dementia
SD: Standard Deviation
TAU: Treatment as usual
WHO-5: Well Being Index Score
